# Antimicrobial efficacy of *Punica granatum Lythraceae* peel extract against pathogens belonging to the ESKAPE group

**DOI:** 10.3389/fmicb.2024.1383027

**Published:** 2024-04-22

**Authors:** Elena Scaglione, Daniela Sateriale, Giuseppe Mantova, Martina Di Rosario, Leonardo Continisio, Mariateresa Vitiello, Caterina Pagliarulo, Roberta Colicchio, Chiara Pagliuca, Paola Salvatore

**Affiliations:** ^1^Department of Molecular Medicine and Medical Biotechnologies, University of Naples Federico II, Naples, Italy; ^2^Department of Science and Technology, University of Sannio, Benevento, Italy; ^3^Department of Public Health, Experimental and Forensic Medicine, University of Pavia, Pavia, Italy; ^4^CEINGE-Biotecnologie Avanzate Franco Salvatore s.c.ar.l., Naples, Italy; ^5^Task Force on Microbiome Studies, University of Naples Federico II, Naples, Italy

**Keywords:** pomegranate peel extract, natural by-product, antimicrobial activity, ESKAPE pathogens, intracellular antimicrobial action, multidrug-resistant bacteria, *in vitro* infection model

## Abstract

The improper use and abuse of antibiotics have led to an increase in multidrug-resistant (MDR) bacteria resulting in a failure of standard antibiotic therapies. To date, this phenomenon represents a leading public health threat of the 21st century which requires alternative strategies to fight infections such as the identification of new molecules active against MDR strains. In the last 20 years, natural extracts with biological activities attracted scientific interest. Following the One Health Approach, natural by-products represent a sustainable and promising alternative solution. Consistently, the aim of the present study was to evaluate the antimicrobial activity of hydro-alcoholic pomegranate peel extract (PPE) against MDR microorganisms belonging to *Enterococcus faecium*, *Staphylococcus aureus*, *Klebsiella pneumoniae*, *Acinetobacter baumannii*, *Pseudomonas aeruginosa,* and *Enterobacter* spp. “ESKAPE” group pathogens. Through semiquantitative and quantitative methods, the PPE showed effective antimicrobial activity against Gram-positive and Gram-negative MDR bacteria. The kinetics of bactericidal action of PPE highlighted that microbial death was achieved in a time- and dose-dependent manner. High concentrations of PPE exhibited antioxidant activity, providing a protective effect on cellular systems and red blood cell membranes. Finally, we report, for the first time, a significant intracellular antibacterial property of PPE as highlighted by its bactericidal action against the staphylococcal reference strain and its bacteriostatic effect against clinical resistant strain in the HeLa cell line. In conclusion, due to its characterized content of polyphenolic compounds and antioxidant activity strength, the PPE could be considered as a therapeutic agent alone or in conjunction with standard antibiotics against challenging infections caused by ESKAPE pathogens.

## 1 Introduction

Since ancient times, natural products have been shown to possess many therapeutic properties in the treatment of various diseases. Over the years, due to the improvement of experimental methodologies, it has been possible to characterize a wide range of bioactive secondary metabolites from both terrestrial and marine sources (Dias et al., 2012). In this context, particular attention was directed toward polyphenols derived from botanical sources, which are excellent sources of biocompounds with beneficial and biological activities. The pharmacological mechanism of polyphenols is strongly related to their antioxidant activity. It is known that polyphenols are able to reduce the level of reactive oxygen species, either by donating electrons to them or by acting as chelating agents (Kaur and Kapoor, 2002). The main beneficial properties of these molecules include anti-inflammatory, anti-allergic, antiatherogenic, antithrombotic, and antimutagenic effects (Ellis et al., 2011). In addition, several studies demonstrate that polyphenols modulate the human immune system by influencing the activation and proliferation of white blood cells as well as the production of cytokines involved in immunological defense (John et al., 2011). Among the different natural compounds studied for their beneficial properties in the medical field, the attention on the bioactive components present in various parts of the *Punica granatum Lythraceae* (*L.*) (pomegranate) plant has increased in recent years (Fakudze et al., 2022). The beneficial effects of the pomegranate fruit reside in the juice but also in secondary components such as the peel, seeds, leaves, and flowers of the tree (Kanatt et al., 2010). Previous findings showed that high polyphenols content in pomegranate juice and peel, including ellagitannins and anthocyanins, has potent antioxidant activity (Pagliarulo et al., 2016; Ferrazzano et al., 2017). Peel itself represents approximately 50% of fruit weight and is reported to contain stronger antioxidant activity compared to the juice (Sood and Gupta, 2015; Pagliarulo et al., 2016), making it a potential source of bioactive compounds. The pomegranate peel has a significant amount of protein, polysaccharides, minerals, and phenolic compounds such as flavonoids, hydrolyzable tannins, and phenolic acids (Teniente et al., 2023). In addition, it has been shown that pomegranate peel extract (PPE) has a variety of biological activities, such as anti-oxidation, antitumor, anti-inflammatory, neuroprotection, antiviral, and antibacterial (Xiang et al., 2022). Pomegranate has a broad spectrum of antimicrobial effects and has an evident inhibitory effect on both Gram-negative and Gram-positive bacteria growth and on eukaryotic microorganism growth such as fungi and molds (Wan et al., 2013; Singh et al., 2019). Recent studies have demonstrated that PPE and aril extracts have a strong antibacterial activity, inhibiting the growth and survival of two clinical isolates of *Staphylococcus aureus* (*S. aureus*) and *Escherichia coli*, with an antibacterial efficacy of the crude peel extract greater than that of the purified (Pagliarulo et al., 2016).

Also of great applicative interest is the antimicrobial and antibiofilm effect demonstrated by pomegranate polyphenolic extracts with important synergy when used in combination with other botanical polyphenolic extracts or in association with antibiotics in *in vitro* adjuvant therapies against cariogenic bacteria (Sateriale et al., 2020a,b). The antibacterial activity of PPE is mainly mediated by changing the structure of cells, inhibition of bacterial biofilm formation, and inhibition of bacterial growth in combination with antibiotics (Xiang et al., 2022).

The antimicrobial properties of pomegranate extracts are of great interest, especially considering the continuing risk of bacterial strains developing resistance to standard antibiotics. Currently, infections associated with multidrug-resistant (MDR) bacteria still represent a great challenge to treat due to limited therapeutic options, thus requiring alternative antimicrobial strategies (World Health Organization (WHO), 2014; Colicchio et al., 2021). Antibiotic resistance is a growing worldwide problem and threatens the important health benefits gained from the development of these drugs. This global crisis reflects the over and improper use of antibiotics in both human and veterinary medicine which has led to an increase in the rate of antimicrobial resistance (World Health Organization (WHO), 2014).

In February 2017, to focus on the research and development of new antibiotics, the World Health Organization (WHO) published a list of pathogens for which the production of new antimicrobials is urgently needed (De Oliveira et al., 2020). In this scenario, six pathogens that exhibit multi-antibiotic resistance mechanisms and high virulence are *Enterococcus faecium*, *S. aureus*, *Klebsiella pneumoniae*, *Acinetobacter baumannii* (*A. baumannii*), *Pseudomonas aeruginosa* (*P. aeruginosa*), and *Enterobacter* spp. that have been called “ESKAPE” microorganisms (Boucher et al., 2009). ESKAPE pathogens are responsible for the majority of nosocomial infections and are able to “escape” to the biocidal action of conventional antimicrobial agents (Founou et al., 2017). For instance, *P. aeruginosa*, *A. baumannii,* and *S. aureus* cause severe infections in susceptible individuals due to several virulence factors that, through sophisticated regulatory mechanisms, allow them to adapt easily to many hostile microenvironments increasing drug resistance to conventional antibacterial agents (Phatthalung et al., 2012; Li and Webster, 2018; Peppoloni et al., 2022). Several studies have demonstrated that pomegranate extract significantly impairs *P. aeruginosa* growth and biofilm formation in a dose-dependent manner (Pang et al., 2019). Moreover, recent data showed that novobiocin treatment in combination with pomegranate extract was significantly effective against *A. baumannii*, an MDR pathogen hard to control (Phatthalung et al., 2012). It was also demonstrated that the pomegranate crude extracts interfered with *S. aureus* growth and survival with time- and dose-dependent effects (Pagliarulo et al., 2016).

Although there is currently a body of literature on the antibacterial properties of polyphenols deriving from pomegranate, this study evaluated, to our knowledge for the first time, the antibacterial activity of PPE, against clinical isolates of methicillin-resistant *Staphylococcus aureus* (MRSA) strain, *P. aeruginosa,* and *A. baumannii* MDR strains compared to reference American Type Culture Collection (ATCC) strains. Our results, obtained from microbiological tests, have highlighted how PPE can represent a reservoir of valuable therapeutic agents that can be used in new sustainable and effective strategies of preventive medicine and adjuvant therapy against infections caused by MDR pathogens that are a world-concerning health problem.

## 2 Materials and methods

### 2.1 Preparation of hydro-alcoholic polyphenolic extracts from peel pomegranate

The extract used in this study was prepared from samples of fresh dry pomegranate peel fruit, harvested from plants growing in the Campania region countryside in the Avellino areas (40.9N, 14.7E, data available on the platform of Global Biodiversity Information Facility, GBIF, https://www.gbif.org/occurrence/3949347683), with solid–liquid solvent extraction method. The dilution ratio was 1:10 (w/v). The hydro-alcoholic polyphenolic extract of pomegranate peel used in this study was prepared according to Pagliarulo et al. (2016), with some modulation. In brief, pomegranate peel powder (5 g) was homogenized in 25 mL of an extraction buffer composed of ethanol (≥99.8% ethanol, CAS 64.17.5, Sigma-Aldrich, Merck, Darmstadt, Germany) and distilled water at 50% (v/v), for 30 min at room temperature in the dark. After centrifugation at 10,000 rpm for 15 min (Centrifuge 5,804 R, Eppendorf, Milan, Italy), the supernatant was filtered by a vacuum filtration unit with a 0.45 μm porosity membrane (Sterilcup^®^ S2HVU02RE Filtration System, Merck-Millipore, Darmstadt, Germany) (Sateriale et al., 2020a). Finally, the characterization of phenolic compounds was obtained by high-performance liquid chromatography (HPLC) (HP 1100, Agilent Technologies, Palo Alto, California, United States) and matrix-assisted laser desorption/ionization time-of-flight mass spectrometry (MALDI-TOF MS) analysis (Voyager DE-Pro^R^, PerSeptive Biosystems, Framingham, Massachusetts, United States).

### 2.2 Bacterial isolates and microbial growth

The antimicrobial activity of *Punica granatum* L. peel hydro-alcoholic extract was evaluated against some pivotal pathogens belonging to the ESKAPE group and against reference strains: *S. aureus* ATCC 6538 (ATCC Manassas, Virginia, United States) and MRSA strain 2 and 3; *P. aeruginosa* ATCC 27853 (ATCC Manassas, Virginia, United States), *P. aeruginosa* MDR strain 1, and *P. aeruginosa* MDR strain 2; *A. baumannii* MDR strain 1 and *A. baumannii* MDR strain 2 (Colicchio et al., 2021). The identification of isolates was performed by MALDI-TOF mass spectrometer (Bruker Daltonics, MALDI Biotyper, Fremont, CA, United States). Bacteria were cultured in aerobic conditions at 37°C in a thermostatic chamber (Misa Sud Refrigerazione, Pomezia, Rome, Italy) in Tryptic Soy Agar (TSA) and Tryptic Soy Broth (TSB) medium (Oxoid S.p.a., Milan, Italy) and Mueller Hinton Agar (MHA) (Oxoid S.p.a., Milan, Italy). The profile of susceptibility to antibiotics was evaluated using the VITEK^®^ 2 bioMérieux System (bioMérieux, Marcy-l’Étoile, France) and was previously reported (Colicchio et al., 2021). The isolates were stored frozen at – 80°C in TSB supplemented with 10% (v/v) glycerol (Carlo Erba Reagents, Milan, Italy) until use, and the working cultures were activated in the respective broth in an orbital incubator shaker (C24 Incubator shaker, New Brunswick Scientific, NJ, United States) at 37°C for 15–18 h.

### 2.3 *In vitro* antibacterial activity assays of the hydro-alcoholic polyphenolic PPE: agar diffusion method

To evaluate the antibacterial effects of the hydro-alcoholic polyphenolic extract from peel pomegranate against ESKAPE group pathogens, an *in vitro* antimicrobial assay was performed using the agar diffusion method (Bauer et al., 1966; Pagliarulo et al., 2018). In brief, bacteria were grown in TSB until they reached an optical density (O.D.) of 0.5 at a wavelength of 600 nm. Then, an aliquot of microbial suspension (200 μL) was spread on the MHA medium, 6-mm blank sterile paper disks (Oxoid S.p.a., Milan, Italy) were placed on agar medium, and then, they were impregnated with different concentrations of extract (from 1 mg/disk to 10 mg/disk). Gentamicin (GEN) [10 μg/disk] (Merck KGaA, Readington, New Jersey, United States), ciprofloxacin (CIP) [10 μg/disk] (Merck Life Science S.r.l., Milan, Italy), and colistin (COL) [10 μg/disk] (Merck Life Science S.r.l., Milan, Italy) were used as a positive control, while the hydro-alcoholic (HA) extraction buffer was used as a negative control. After the plates’ incubation in a thermostatic chamber at 37°C for 24 h, the size of inhibition zones was measured. The diameter of the inhibition zones (expressed in mm) produced by the natural extract allowed for the expression of its *in vitro* antimicrobial activities against the selected microorganisms. All experiments were performed in triplicate, with three independent cultures; the results obtained were analyzed and graphically reported by using “GraphPad Prism 6” software. The results are presented as mean ± SD. The statistical significance was determined using Student’s *t*-test (*p*-value ≤0.05).

### 2.4 *In vitro* antibacterial activity assays of the hydro-alcoholic polyphenolic PPE: broth dilution method

The susceptibility of ESKAPE pathogens to different concentrations of polyphenolic PPE was determined by the tube dilution method with a standard inoculum of 1×10^5^ colony forming units/mL (CFU/mL), according to the Clinical and Laboratory Standards Institute (CLSI) 2017 guidelines (Varaldo, 2002; Clinical and Laboratory and Standard Institute, 2020). The extracts were added to achieve a final concentration of 2, 4, 8, 10, 12, 16, 20, and 25 𝜇g/𝜇L, for *S. aureus* ATCC 6538, MRSA strain 2 and strain 3, and *A. baumannii* MDR strain 1 and strain 2. For *P. aeruginosa* ATCC 27853 and *P. aeruginosa* MDR strain 1 and strain 2, a final concentration of 10, 20, 30, 40, 50, 60, 70, and 80 𝜇g/𝜇L was used. The samples were incubated in a thermostatic chamber at 37°C for 24 h. GEN, CIP, and COL were used as a positive control, while the HA extraction buffer was used as a negative control. Minimum inhibitory concentration (MIC) was assigned to the lowest concentration of PPE that prevents microbial growth, through the O.D._600nm_ evaluation. The minimal bactericidal concentration (MBC) was defined as the minimum concentration of natural extract that killed 99% of bacteria from the initial inoculum, by the CFU method.

### 2.5 Time-killing assay with PPE

To verify the effect of PPE on selected ESKAPE group pathogens, survival assays were performed in the presence of increasing concentrations of the extract ranging from 8 μg/μL to 80 μg/μL. To evaluate the fitness of each strain, during the observation period (72 h), aliquots of bacterial suspensions were taken, and 10-fold serial dilutions were spread on TSA and incubated in a thermostatic chamber at 37°C for 24–48 h to evaluate viable counts using the CFU method. All experiments were performed in triplicate, with three independent cultures, and the results obtained were analyzed and graphically reported by using “GraphPad Prism 6” software. The results are presented as mean ± SD. The statistical significance was determined using Student’s *t*-test (*p*-value ≤0.05).

### 2.6 Cell viability studies

HeLa (ATCC CCL-2; human epithelial cell) and HEK-293 cells (ATCC CRL-1573; human embryonic kidney cell) stored in liquid nitrogen were thawed by gentle agitation for 2 min in a water bath (Type M408-BM, MPM Instruments S.r.l., Italy) at 37°C. After thawing, cells were grown in Dulbecco’s modified Eagle’s minimal essential medium (DMEM) (Microgem, Naples, Italy), supplemented with 10% (v/v) fetal bovine serum (FBS) (Microgem), 2 mM L-glutamine (Himedia, Mumbai, India), and antibiotic solution 100X liquid with 10,000 U penicillin and 10 mg streptomycin (Himedia, Mumbai, India). The cells were incubated for 24 h at 37°C with 5% CO_2_ (HERA Cell 150, Heraeus, Thermo Fisher Scientific, United States) to allow them to grow and form a monolayer. Cells grown to 80% confluency were washed with phosphate-buffered saline (PBS), trypsinized with 3 mL of trypsin–EDTA solution 1× (Himedia, Mumbai, India) diluted, counted, and seeded (5x10^4^cells/200 μL per well) into a 96-well microtiter tissue culture plates (Falcon 96 well, REF 353072, Corning Incorporated, United States) for 24 h in triplicate. The reduction in the proliferation of the cells was evaluated by the 3-[4.5-dimethylthiazol-2-yl]-2.5-dipheniltetrazolium bromide (MTT) (CAS 298.93.1, Sigma-Aldrich) assay which enables the measurement of metabolic changes by using a microplate reader (A_570 nm_) (Sunrise^™^, TECAN, Switzerland). The cells were incubated with or without increasing concentrations of PPE (50, 100, and 150 μg/μL) for 24 and 30 h at 37°C with 5% CO_2_. Adherent cells were stained with MTT dye solution according to the manufacturer’s instructions and incubated for 4 h. After incubation, the presence of the violet crystals that normally indicate metabolization of MTT was verified. Then, the medium was removed and 180 μL of dimethyl sulfoxide (Sigma-Aldrich) was added to dissolve the MTT crystals. The eluted specific stain was measured with a microplate reader. The proliferation index of the treated cells was compared to that of the negative control (cell plus medium without PPE). The cellular viability was determined using the following formula:


$$\mathrm{Cellular}\;\mathrm{viability}\;\left(\%\right)=\left(\frac{\mathrm{Absorbance}\;\mathrm{of}\;\mathrm{treated}\;\mathrm{cells}}{\mathrm{Absorbance}\;\mathrm{of}\;\mathrm{control}\;\mathrm{cells}}\right)*100$$

Values are mean ± standard deviation (SD) of triplicate experiments. Data were expressed as the mean ± SD. The statistical significance was determined using Student’s *t*-test (*p*-value ≤0.05). The results obtained were graphically reported using “GraphPad Prism 6” software.

Reactive oxygen species (ROS) production was evaluated using the 2′,7′-dichlorodihydrofluorescein diacetate (DCFH-DA) assay according to Arkoub-Hamitouche et al. (2020). This assay is based on the oxidation of the non-fluorescent compound 2′,7′-dichlorohydrofluorescein (DCFH) into the fluorescent compound dichlorofluorescein (DCF) in the presence of ROS. In detail, to test the effect of PPE on intracellular ROS production, concentrations of 50, 100, and 150 μg/μL of extract were added to 3×10^4^ HeLa cells in a 96-well microtiter tissue culture plates and incubated at 37°C with 5% CO_2_ for 24 h. Moreover, the cells were treated with H_2_O_2_ 3.6% (Hawai Srl Unipersonale, PR, Italy) diluted in DMEM 25% (v/v) and with medium alone, used as a positive and negative control, respectively.

At the end of exposure time, cells were stained with 10 μM DCFH-DA dye and incubated in the dark at 37°C for 30 min. Then, cells were washed twice using PBS(1X), and the fluorescence emission measurement was performed in PBS(1X) using a fluorescence microplate reader (BioTek Synergy H1, Agilent Technologies, Inc.) with an excitation wavelength of 485 nm and emission wavelength of 530 nm. Values are expressed as a percent relative to the control condition. Data obtained from three independent experiments were expressed as the mean ± SD. The statistical significance was determined using Student’s *t*-test (*p* ≤ 0.05). The results obtained were graphically reported using “GraphPad Prism 6” software.

### 2.7 Hemolysis inhibition assay

The inhibition of human erythrocyte lysis by PPE was evaluated following Nabavi et al. (2012) method with some modifications. In detail, hemolysis was induced using hydrogen peroxide (H_2_O_2_) as a free radical initiator. The whole human blood, collected from a healthy volunteer, was centrifuged at 500 × *g* for 5 min at room temperature (Multifuge 1 S-R, Heraeus, Thermo Fisher Scientific, United States) to isolate erythrocytes. The cells were washed two times with 150 mM NaCl solution, and subsequently, erythrocytes were resuspended in an equal volume of PBS(1X). The sample was capped and inverted a few times to gently mix and was centrifuged at 500 × *g* for 5 min and resuspended in an equal volume of PBS(1X); then, a final suspension of 5% (v/v) of washed erythrocytes in PBS(1X) was prepared. To 100 μL of a 5% (v/v) suspension, aliquots of 50 μL containing different concentrations of PPE (50, 100, and 150 μg/μL) were added. Then, 100 μL of H_2_O_2_ 3.6% (Hawai Srl Unipersonale, PR, Italy) was added in each reaction. Samples were shaken gently during incubation for 1 h in a thermostatic chamber at 37°C. After incubation, the samples were centrifuged at 500 × *g* for 5 min at room temperature, and the supernatants were transferred in a transparent flat-bottom 96-well plate (Micro test plate, 96 well, Sarstedt, Germany) to determine the extent of hemolysis by measuring optical density at a wavelength of 450 nm using a microplate reader (Plate reader A4, das S.r.l., Italy). Likewise, the erythrocytes were treated with H_2_O_2_ without PPE to obtain complete hemolysis, and PBS(1X) was used as a negative control. The inhibition of hemolysis was calculated according to the formula:


$$\mathrm{Inhibition}\;\mathrm{of}\;\mathrm{Hemolysis}\;\left(\%\right)=100-\left(\frac{\begin{array}{l} A_{450\mathrm{nm}}\;PPE\;\mathrm{treatment}\\ -A_{450\mathrm{nm}}PBS\left(1X\right) \end{array}}{\begin{array}{l} A_{450\mathrm{nm}}H_{2}O_{2}\\ -A_{450\mathrm{nm}}PBS\left(1X\right) \end{array}}\right)*100$$

All experiments were performed in triplicate, and the results obtained were analyzed and graphically reported by using “GraphPad Prism 6” software. The results are presented as mean ± SD. The statistical significance was determined using Student’s *t*-test (*p*-value ≤0.05).

### 2.8 Invasion assays and protection in HeLa cells with hydro-alcoholic polyphenolic PPE

*S. aureus* ATCC 6538 and MRSA strain 2 invasion assays were performed as described previously (Colicchio et al., 2015, 2021). For standard invasion and intracellular viability assays, HeLa cells were grown in DMEM with 2 mM L-glutamine and 10% heat-inactivated FBS. Confluent cell monolayers were infected for 1 h at a multiplicity of infection (MOI) of 100 for both bacterial strains and then exposed to GEN (Sigma-Aldrich) to kill the remaining extracellular bacteria. GEN treatment was performed using 50 μg/mL, a concentration 10-fold the MIC for 30 min at 37°C with 5% CO_2_ (Binder C150, Binder, Germany) (Colicchio et al., 2021). Thereafter, cells were extensively washed with PBS(1X) to remove GEN and dead extracellular bacteria. For the quantification of intracellular staphylococci, HeLa cells were destroyed using 0.5% saponin (CAS 8047.15.2, Sigma-Aldrich). The lysed cell suspension was plated on TSA, and the numbers of CFU were counted the day after. When required, cells were re-incubated in the cell culture medium at various times (5, 10, 24, and 30 h) to evaluate the intracellular antimicrobial activity of hydro-alcoholic PPE after GEN treatment. The extract was added to the samples with increasing concentration (50, 100, and 150 μg/μL) for 5, 10, 24, and 30 h and subsequently washed twice with PBS(1X), and finally, cells were destroyed using saponin. In all experiments, the bacteria were centrifuged (60 × g) onto cells to start the assay. The experiments were performed in triplicate, and data were expressed as the mean ± SD. The statistical significance was determined using Student’s *t*-test (*p*-value ≤0.05). The results obtained were graphically reported using “GraphPad Prism 6” software.

## 3 Results

### 3.1 *In vitro* antibacterial activity of the hydro-alcoholic PPE

**Figure 1:**
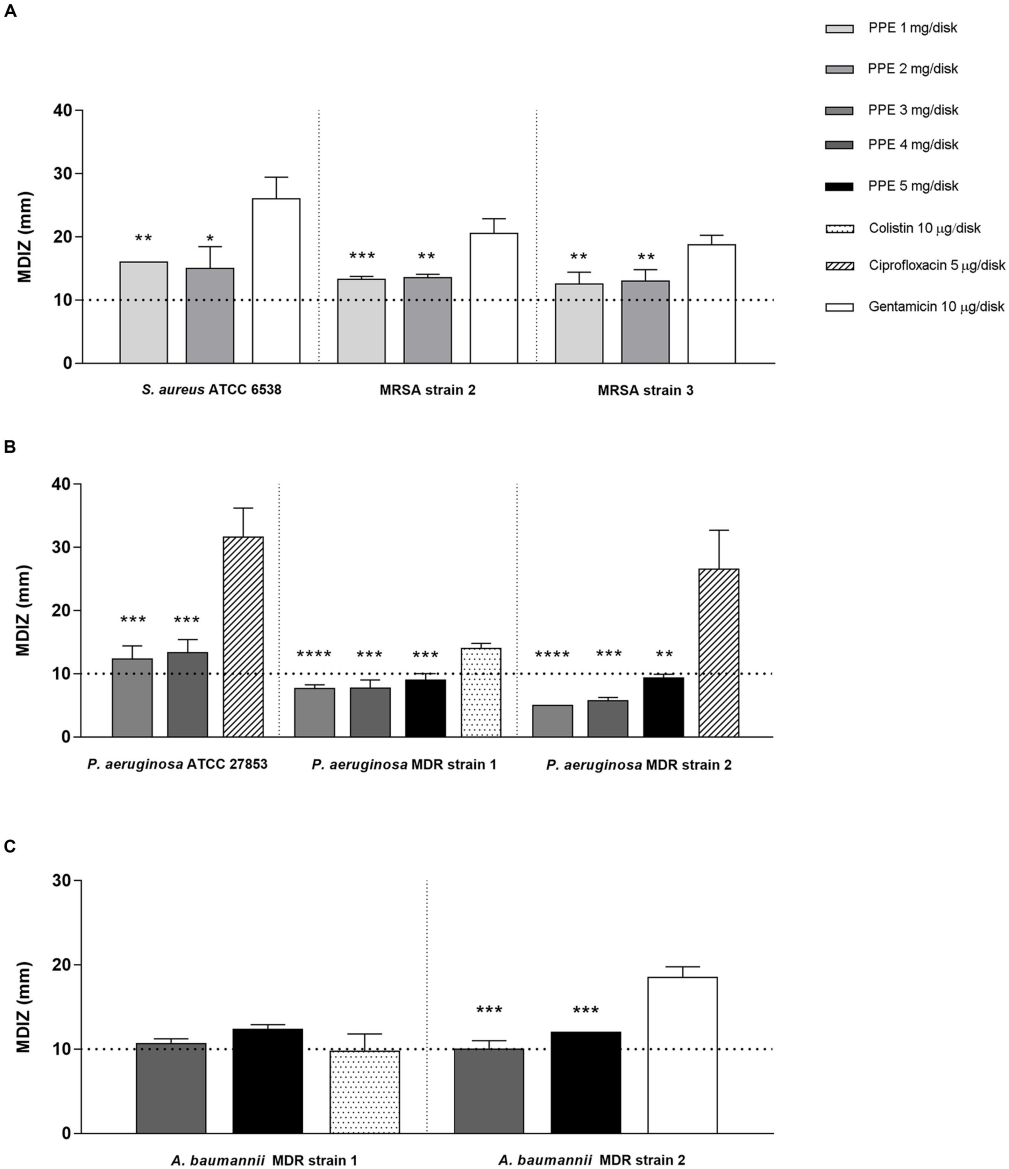
*In vitro* antibacterial activity of PPE by the agar well diffusion method. Results of agar diffusion method of PPE against *S. aureus* ATCC 6538 and MRSA strains **(A)**, *P. aeruginosa* ATCC 27853 and MDR strains **(B)**, and *A. baumannii* MDR strains **(C)**. Graphical representation of the results; the MDIZ in mm is reported as the mean of values obtained from assays in triplicate ± SD. Statistical significance was examined using Student’s *t*-test. Asterisks indicate the statistical significance with respect to the positive control (**** *p* < 0.0001; *** *p* < 0.001; ** *p* < 0.01; * *p* < 0.05).

The antimicrobial properties of hydro-alcoholic polyphenolic PPE were evaluated against reference strains and clinical isolates exhibiting an appreciable inhibitory activity against ESKAPE group pathogens. The mean diameters of the inhibition zones (MDIZ) of bacterial growth exerted by the extract against ESKAPE group pathogens are shown in Figure 1. Previously, the antibacterial activity of pomegranate extracts determined by the agar diffusion method was considered effective for values equal to or greater than 10 mm in diameter (Pagliarulo et al., 2016). For staphylococci isolates, the MDIZs at 1 mg/disk were 13.25 ± 0.5 mm for MRSA strain 2 and 12.5 ± 1.9 mm for MRSA strain 3, while for 2 mg/disk tested concentration, the MDIZs were 13.5 ± 0.5 mm for MRSA strain 2 and 13 ± 1.8 mm for MRSA strain 3 (Figure 1A). For the reference *S. aureus* ATCC 6538 strain, the MDIZs for both tested concentrations were, respectively, 16 ± 0 mm (1 mg/disk) and 15 ± 3.4 mm (2 mg/disk) (Figure 1A). For *P. aeruginosa* MDR 1 and MDR 2 strains, extract concentrations ranging between 3 and 5 mg were tested, achieving the greatest inhibition diameter at 5 mg/disk, respectively, of 9 ± 1 mm and 9.3 ± 0.5 mm (Figure 1B). The reference strain *P. aeruginosa* ATCC 27853 exhibited at the higher tested concentration a MDIZ of 13.3 ± 2 mm (4 mg/disk) (Figure 1B). For *A. baumannii* MDR 1 and MDR 2 strains, the extract efficiency was observed at the lowest tested concentration, with MDIZ values, respectively, of 10.6 ± 0.5 mm and 10 ± 1 mm (4 mg/disk) (Figure 1C). In addition, for 5 mg/disk concentration, the MDIZ value was 12.3 ± 0.5 mm for MDR strain 1 and 12 ± 0 mm for MDR strain 2 (Figure 1C). The inhibition zone (mm) is reported as the mean of triplicate assay ± SD. GEN, CIP, and COL were tested as positive control, showing antibacterial efficacy against all tested strains, while no effects were observed with the HA buffer used for the extraction as a negative control (data not shown).

The antimicrobial activity of PPE against ESKAPE group pathogens was also quantitatively evaluated by the dilution tube method, according to the CLSI guidelines (CLSI, 2015). For *A. baumannii* MDR strains, the MIC value of PPE was 10 μg/μL, and the MBC value was 16 μg/μL (Table 1). The PPE exhibited a MIC value of 20 μg/μL for *P. aeruginosa* MDR 1 and 30 μg/μL for *P. aeruginosa* ATCC and *P. aeruginosa* MDR 2, while the MBC value for *P. aeruginosa* MDR strain 1 and 2 was 40 μg/μL, and 60 μg/μL for *P. aeruginosa* ATCC (Table 1). The hydro-alcoholic PPE exerted a strong antibacterial effect also against *S. aureus* ATCC and MRSA strains with a MIC/MBC value ranging from 4 to 12 μg/μL (Table 1). All bacteria tested in this study were sensitive to at least one antibiotic tested as a positive control.

**Table 1:** Antimicrobial activities of PPE against ESKAPE pathogens group.

Isolate	PPE (μg/μL)		GEN^a^ (μg/mL)		CIP^b^ (μg/mL)		COL^c^ (μg/mL)	
	MIC^d^	MBC^e^	MIC	MBC	MIC	MBC	MIC	MBC
*Acinetobacter baumannii* MDR^g^ strain 1	10	16	^f^NT	NT	NT	NT	10	10
*Acinetobacter baumannii* MDR strain 2	10	16	2	2	NT	NT	NT	NT
*Pseudomonas aeruginosa* ATCC^h^ 27853	30	60	NT	NT	0.25	4	NT	NT
*Pseudomonas aeruginosa* MDR strain 1	20	40	NT	NT	NT	NT	10	10
*Pseudomonas aeruginosa* MDR strain 2	30	40	NT	NT	0.25	2	NT	NT
*Staphylococcus aureus* ATCC 6538	4	4	5	5	NT	NT	NT	NT
*Staphylococcus aureus* MRSA^i^ strain 2	12	12	5	5	NT	NT	NT	NT
*Staphylococcus aureus* MRSA strain 3	4	8	5	5	NT	NT	NT	NT

a GEN, gentamicin.

b CIP, ciprofloxacin.

c COL, colistin.

d MIC, minimum inhibitory concentration values are expressed as concentration μg/μL of extract or as concentration μg/mL of antibiotics.

e MBC, minimum bactericidal concentration values are expressed as concentration μg/μL of extract or as concentration μg/mL of antibiotics.

f NT, No tested.

g MDR, multidrug-resistant.

h ATCC, american type culture collection.

i MRSA, methicillin-resistant *Staphylococcus aureus*.

### 3.2 Effects of PPE on bacterial survival by time-killing assay

**Figure 2:**
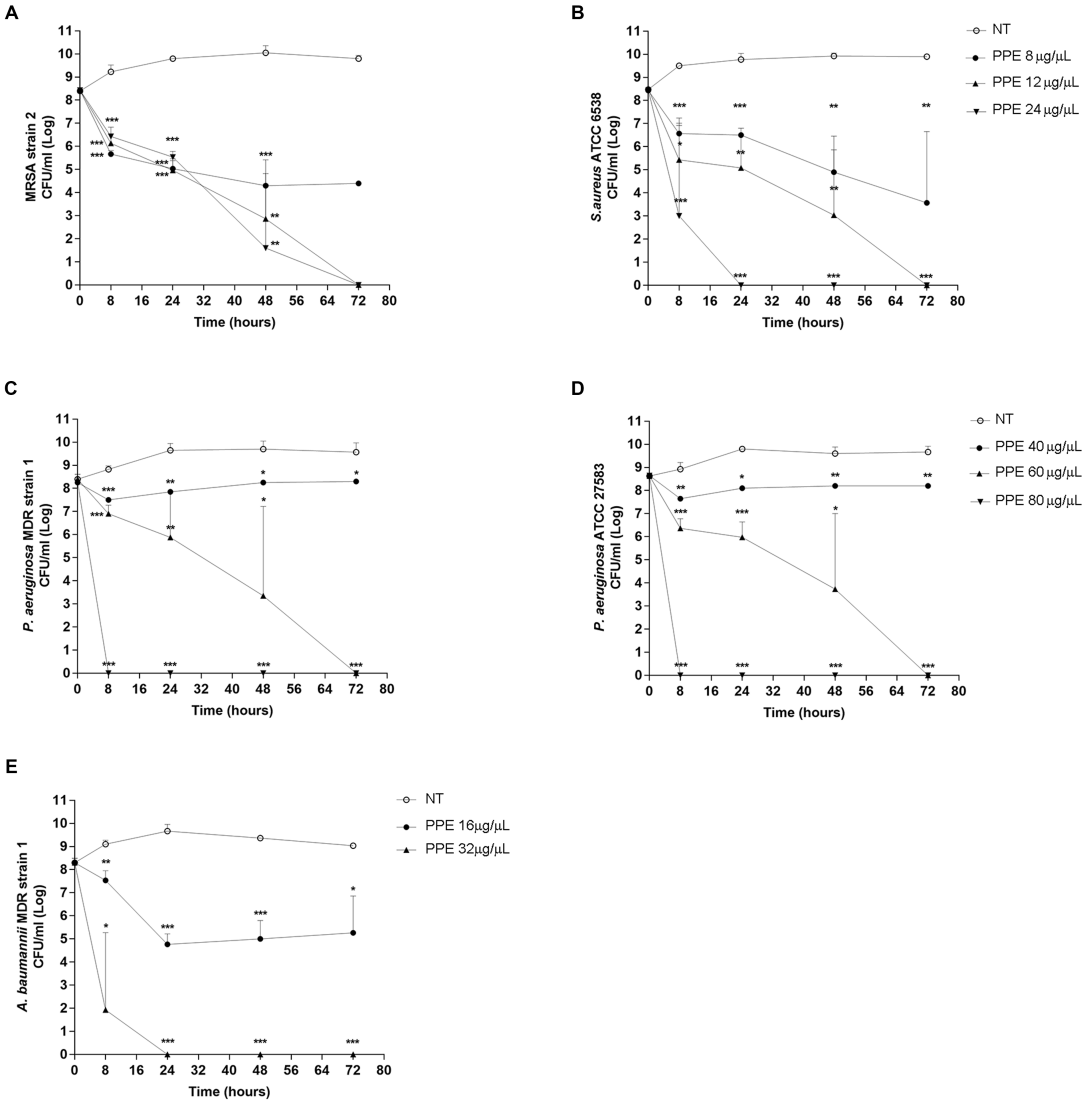
Time-killing assay of PPE on ESKAPE group pathogens. Time-killing curve of PPE at different concentrations against MRSA strain 2 and *S. aureus* ATCC 6538 **(A,B)**, *P. aeruginosa* MDR strain 1 and *P. aeruginosa* ATCC 27853 **(C,D)**, and *A. baumannii* MDR strain 1 **(E)**. NT indicates untreated control. Statistical significance was examined using Student’s *t*-test. Data represent mean values of three independent experiments, and asterisks denote values statistically significant (*** *p* < 0.001; ** *p* < 0.01; * *p* < 0.05).

To verify the effect of PPE on the fitness of selected pathogens, the survival rate of each bacterial strain was evaluated for 72 h, with increasing concentrations of hydro-alcoholic PPE. Evaluation of viable counts showed a great bactericidal activity of PPE against ESKAPE pathogen fitness and against reference strains used as a control. As shown in Figure 2A, MRSA strain 2, when exposed at a concentration of PPE of 12 μg/μL, exhibited a significant survival reduction after 8 h of treatment, achieving bacterial cell death at the end of the observation time. Moreover, after the exposure to 24 μg/μL of PPE, the same trend has been recorded. Similarly, with 12 μg/μL of extract, the same killing kinetics over time was observed for the *S. aureus* susceptible reference strain with a bactericidal effect at 72 h (Figure 2B). In addition, the exposure to 24 μg/μL caused a significant reduction in the first 8 h of observation, reaching bacterial death already at 24 h (Figure 2B). A bacteriostatic effect was observed for both staphylococci strains when treated with 8 μg/μL of PPE (Figures 2A,B). Interestingly, for *P. aeruginosa* MDR strain 1 strain and *P. aeruginosa* ATCC 27853 reference strain, a reduction in bacterial viability over time was constantly observed, up to obtaining a bactericidal action at 72 h, upon exposure to 60 μg/μL of PPE (Figures 2C,D). Furthermore, with a concentration of 80 μg/μL PPE, a bactericidal effect was observed already after 8 h of exposure (Figures 2C,D), and a slight reduction of bacterial viability for both *P. aeruginosa* strains was already observed with 40 μg/μL of PPE (Figures 2C,D). Finally, for the *A. baumannii* MDR strain 1, the assay showed a strong bactericidal activity after 24 h of treatment with 32 μg/μL of PPE, while a reduction in bacterial viability over time was observed even at concentrations of PPE corresponding to 16 μg/μL (Figure 2E). Overall, these findings clearly indicate that the hydro-alcoholic PPEs are able, *in vitro*, to effectively antagonize the growth both of gram-positive and gram-negative ESKAPE group bacteria.

### 3.3 Evaluation of PPE on cell viability

**Figure 3:**
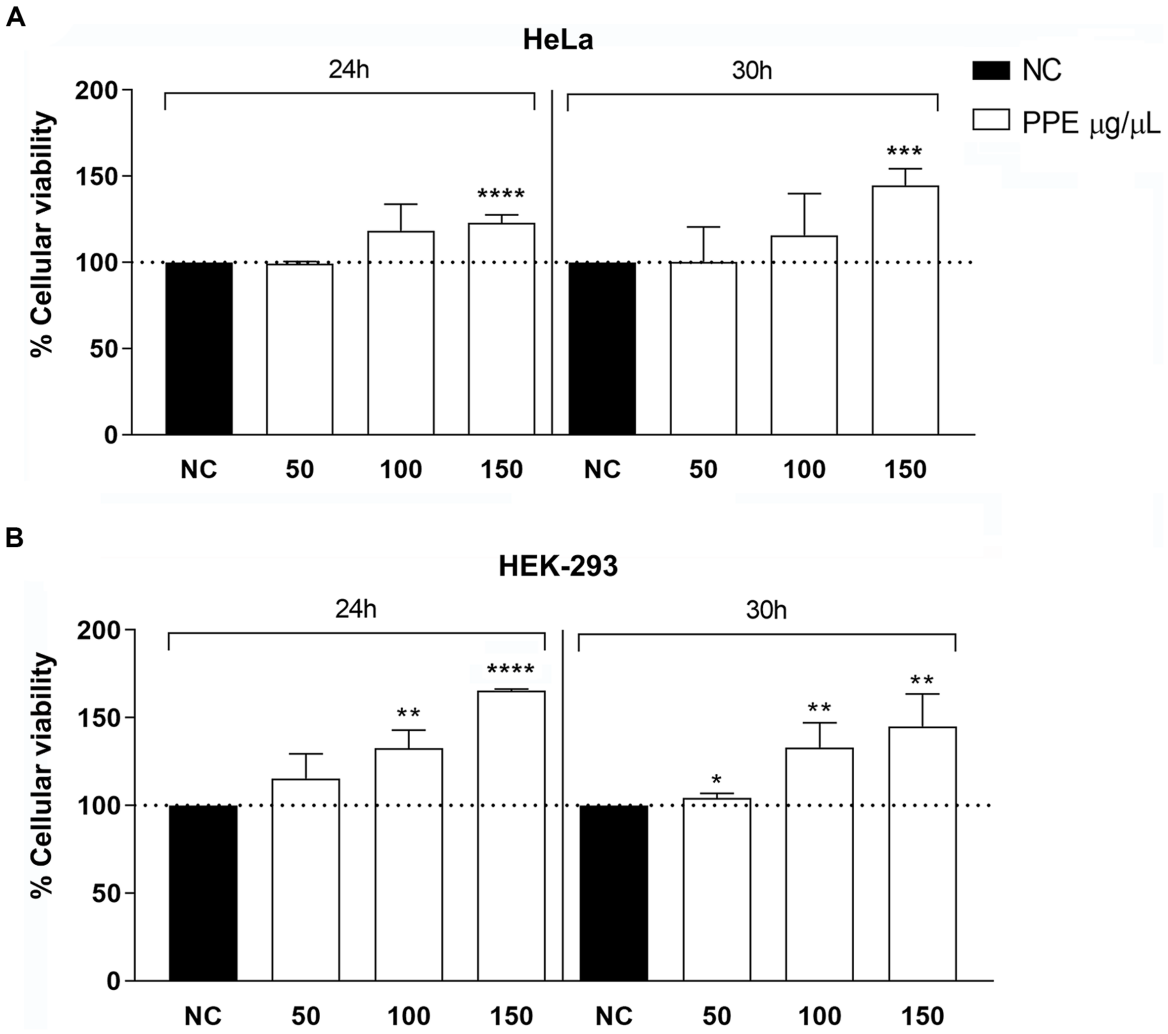
Evaluation of cytotoxicity activity of PPE on HeLa and HEK-293 cells. Effects of 50, 100, and 150 μg/μL of extract for an exposure time of 24 and 30 h on HeLa **(A)** and HEK-293 **(B)** cell lines. The data are expressed as the percentage of absorbance at 570 nm relative to the control. The data are expressed as the mean ± SD of triplicate experiments. NC, negative control. Statistical significance was examined using Student’s *t*-test, and asterisks denote values statistically significant (**** *p* < 0.0001; *** *p* < 0.001; ** *p* < 0.01; * *p* < 0.05).

The cytotoxic activity of the PPE was tested on HeLa and HEK-293 cells at concentrations of 50, 100, and 150 μg/μL for an exposure time of 24 h and 30 h. The PPE was not toxic at all tested concentrations for the selected exposure times (Figure 3). For concentrations of 50 μg/μL of PPE, cell viability was approximately 100% as the negative control at 24 h and 30 h of treatments, for both cell lines. Moreover, after the exposure to 100 μg/μL of PPE, a slight increase in cell viability was observed for all times tested, whereas, with 150 μg/μL of PPE, both cell lines exhibited a significantly increased viability compared to the negative control. Therefore, this experiment shows that the PPE does not induce relevant cytotoxic effects *in vitro*, when used at concentrations ranging from 50 to 150 μg/μL up to 30 h of exposition in HeLa and HEK-293 cell lines.

**Figure 4:**
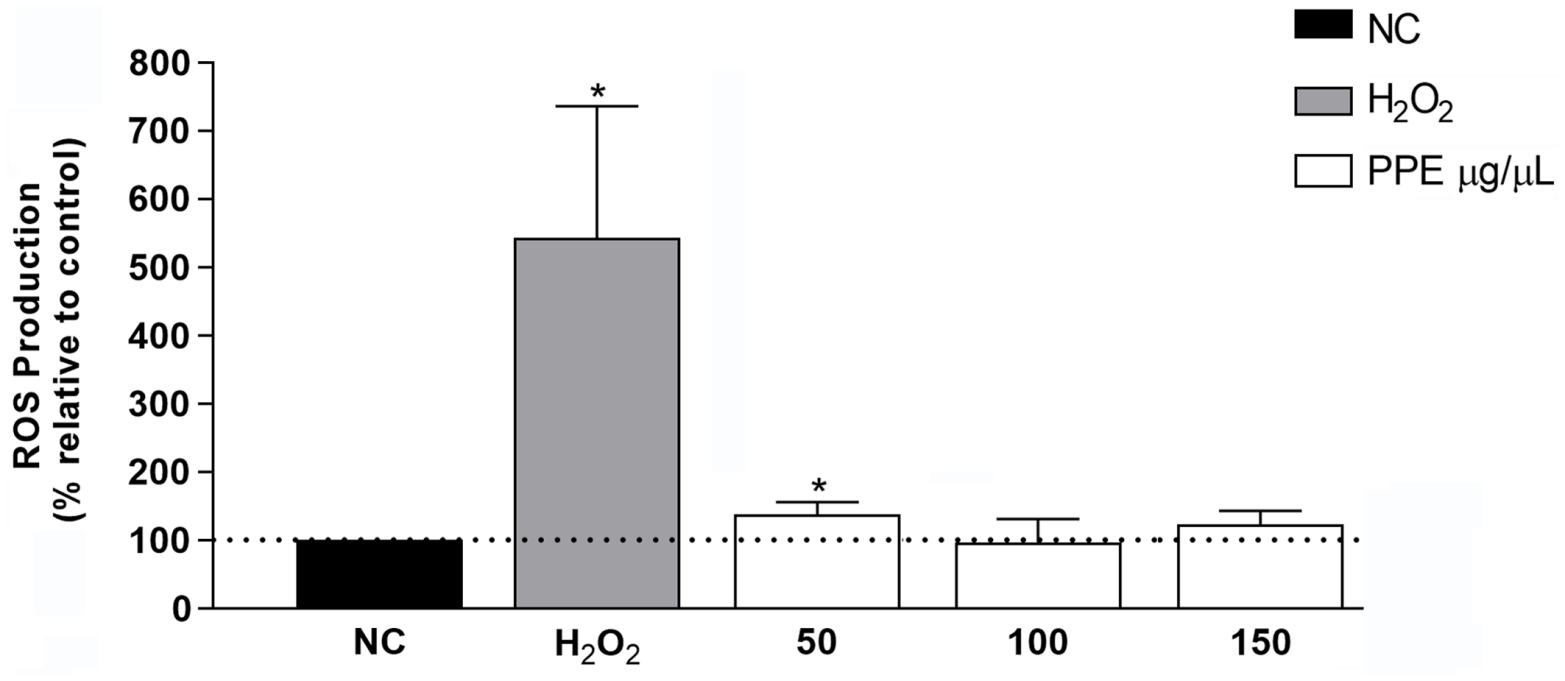
Effect of PPE on intracellular ROS production in the HeLa cell line. Intracellular ROS production induced by 50, 100, and 150 μg/μL of PPE after 24 h of exposure in HeLa cells. The data are expressed as a percent relative to negative control (NC) ± SD of triplicate experiments. H_2_O_2_ was used as a positive control. Statistical significance was examined using Student’s *t*-test, and asterisks denote values statistically significant (*p < 0.05).

To evaluate the effect of PPE on intracellular ROS production, ranging concentrations of PPE (50 to 150 μg/μL) were used to treat the HeLa cell line for 24 h. The PPE did not significantly increase intracellular ROS production with all tested concentrations compared to untreated cells, used as a negative control (Figure 4). Conversely, treatment with H_2_O_2_, used as a positive control, caused a 5-fold increase in intracellular ROS production compared to the negative control, suggesting that PPE may not represent a source of intracellular stress.

**Figure 5:**
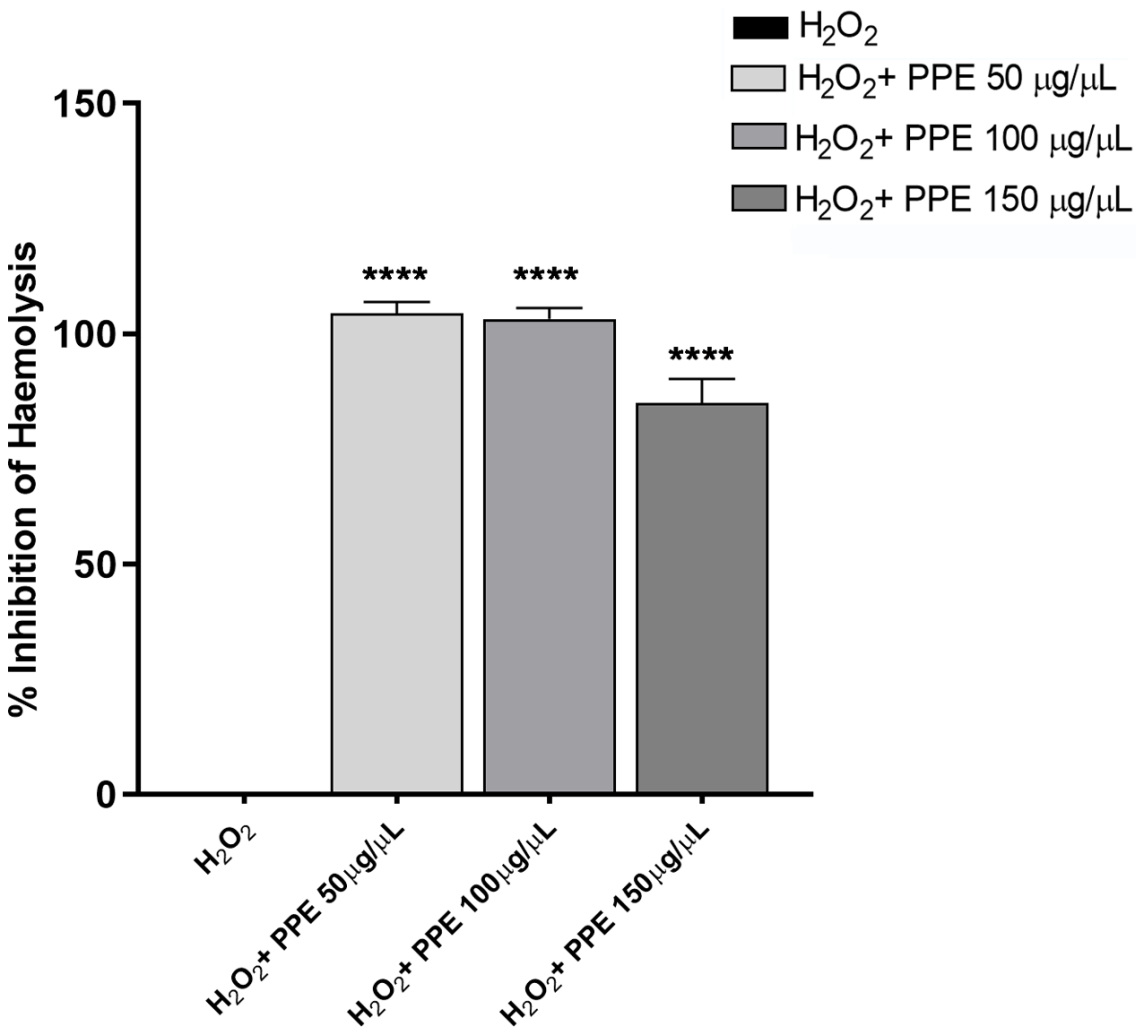
Evaluation of anti-hemolytic activity of PPE. The PPE showed a protective effect against the hemolytic activity of H_2_O_2_ with a percentage of inhibition hemolysis of 104.4% ± 2.5 at 50 μg/μL, 103.2% ± 2.3 at 100 μg/μL, and 85% ± 5.1 with 150 μg/μL. The data are compared to H_2_O_2_ used as a positive control and to the PBS(1X) used as a negative control. Absorbance was read at 450 nm. The data are expressed as the mean ± SD of triplicate experiments, and statistical significance was examined using Student’s *t*-test. Asterisks indicate statistical significance (**** *p* < 0.0001).

To investigate the anti-hemolytic action of PPE, the protective effect of pomegranate extract against the hemolytic activity of H_2_O_2_ solution on human-washed red blood cells was examined. Significant inhibition was found when the cells were treated with PPE after 60 min of incubations, the time necessary to hemolyze 100% of blood cells in the positive control. In detail, PPE showed a statistically significant anti-hemolytic potency at 50 μg/μL and 100 μg/μL, with a percentage of inhibition hemolysis of 104.4% ± 2.5 and 103.2% ± 2.3, respectively (Figure 5), while 85% ± 5.1 of inhibition hemolysis rate was observed with 150 μg/μL of PPE (Figure 5).

All *in vitro* tests demonstrated that PPE does not induce neither cytotoxic effect nor intracellular stress on human cell lines and has high anti-hemolytic activity.

### 3.4 Antimicrobial activity of PPE in intracellular infection system

**Figure 6:**
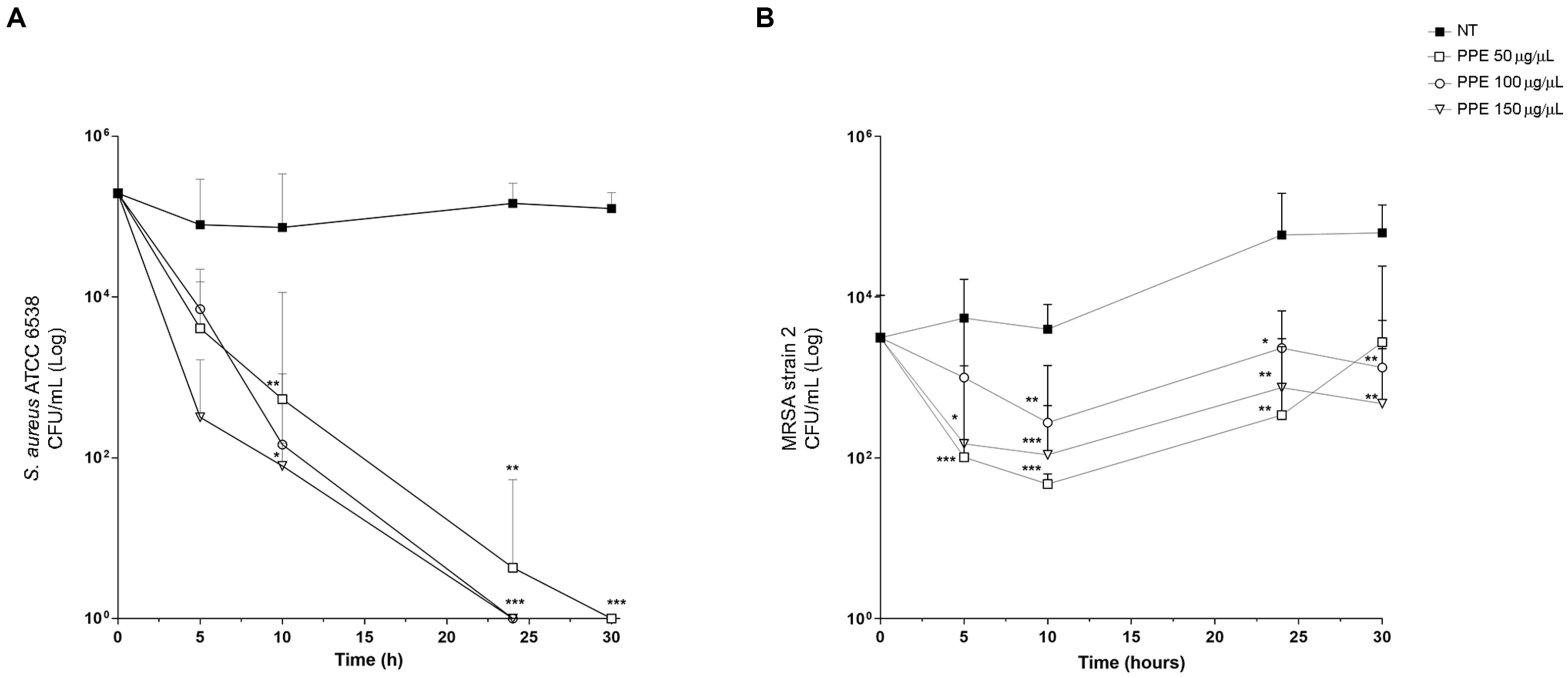
Antibacterial activity of PPE in HeLa cells. Survival assay of intracellular *S. aureus* ATCC 6538 and MRSA strain 2 in HeLa human cells after PPE treatment. HeLa cells (10^5^ cells/well) were infected with staphylococci: **(A)** *S. aureus* ATCC and **(B)** MRSA strain 2 at a multiplicity of infection of 100 treated with GEN and then with PPE at different concentrations or not treated (NT) and re-incubated in Dulbecco’s modified Eagle’s minimal essential medium for the indicated times. After saponin lysis, the numbers of intracellular CFU were recorded. Values are means from at least three independent experiments. The results are shown as the relative number of CFU per well ±SD, and statistical significance was examined using Student’s *t*-test. Asterisks indicate statistical significance (*** *p* < 0.001; ** *p* < 0.01; * *p* < 0.05).

To evaluate whether PPE has an antibacterial activity also in the eukaryotic cell system, invasion assays in HeLa cells with *S. aureus* ATCC 6538 and MRSA strain 2 were performed. For this purpose, after 1 h of infection followed by GEN treatment, the HeLa cells were subsequently exposed to increasing concentrations (50, 100, and 150 μg/μL) of PPE for 5, 10, 24, and 30 h. Then, the number of viable intracellular bacteria was determined by the CFU method. The results demonstrated a strong antibacterial activity of PPE against *S. aureus* ATCC strain in the intracellular compartment (Figure 6A). A constant and increasing reduction of viable intracellular bacteria was observed in a time- and dose-dependent manner, reaching a bactericidal action at concentrations of 100 μg/μL and 150 μg/μL after 24 h of incubation and after 30 h with 50 μg/μL of extract.

For the MRSA strain 2, the 50 μg/μL concentration of PPE showed a slight antibacterial activity at 5 h and 10 h of observation, decreasing its efficacy at the 24-h and 30-h exposure times (Figure 6B), while a constant inhibition of bacterial replication in a dose-dependent manner was observed for 100 μg/μL and 150 μg/μL, at each analyzed time point (Figure 6B).

These data suggest that the PPE was able to efficiently internalize into human cells and exhibits a strong bactericidal or bacteriostatic activity also during the intracellular phase of invasion.

## 4 Discussion

Infectious diseases have been defined as the main challenge to human progress and survival. Furthermore, MDR bacteria are one of the leading public health threats of the 21st century. It is estimated that the spread of antimicrobial resistance will kill 10 million people per year by 2050 representing an urgent issue requiring a global and coordinated action plan to address (Morens et al., 2004; Antimicrobial Resistance Collaborators, 2022). The advent of a growing number of MDR bacteria has several implications that concern not only the wellbeing but also the economic aspect, strongly influencing the costs for the use of drugs and the prolongation of hospitalization times (Aslam et al., 2018). In recent years, with the aim to face the diffusion of MDR bacteria, there has been an increased interest in natural extracts, with biological activities and health advantages.

In this context, several research studies, published in the last 20 years (Singh et al., 2023), highlighted that the fruit of the pomegranate, *Punica granatum L.*, possesses various pharmacological properties including antioxidant, anti-inflammatory, anti-cancer, and antimicrobial activities. Furthermore, PPE is a nutrient by-product of the fruit that is becoming abundant due to an exponential increase in the production of pomegranate products (Argov-Argaman et al., 2020). PPE contains the main active biological compounds to a greater extent than the edible parts of the pomegranate fruit and is known to possess beneficial bioactive compounds, such as the rich variety of antioxidants and phytochemicals (Pagliarulo et al., 2016; Guerrero-Solano et al., 2021).

In this study, we evaluated the antimicrobial action of PPE against MDR microorganisms belonging to the “ESKAPE” group pathogens, responsible for infections that are difficult to treat with standard antibiotics.

Semiquantitative evaluations highlighted an effective antibacterial activity of PPE against *S. aureus* ATCC 6538, MRSA strains 2 and 3, *P. aeruginosa* ATCC 27856, *P. aeruginosa* MDR strains 1 and 2, and *A. baumannii* MDR strains 1 and 2, with MDIZ ranging from 9 to 16 mm.

The antibacterial efficacy of PPE was further confirmed with the microdilution assay, which identified reduced values of MIC and MBC. In particular, for staphylococcal reference and resistant strains and for *A. baumannii* MDR strain, MIC and MBC values ranged from 4 to 12 μg/μL and from 4 μg/μL to 16 μg/μL, respectively, while, for the *P. aeruginosa* reference and MDR strains, the MIC and the MBC values were ranging from 20 μg/μL to 30 μg/μL and from 40 μg/μL to 60 μg/μL, respectively.

The kinetics of bactericidal action of PPE highlighted that microbial death, after exposure to the natural extract, has been achieved in a time- and dose-dependent manner. In detail, the survival of the staphylococcal reference strain was completely abolished in 24 h with 24 μg/μL of PPE and 72 h with 12 μg/μL of the extract, whereas the death of MRSA strain 2 occurred in 72 h after exposure to 24 μg/μL of PPE. Otherwise, with Gram-negative strains, the PPE bactericidal action has been obtained in 8 h with a concentration of 80 μg/μL for both *P. aeruginosa* ATCC strain and *P. aeruginosa* MDR strain 1, 24 h with 32 μg/μL of natural extract for *A. baumannii* MDR strain 1, and 72 h with 60 μg/μL of PPE for both strains of *P. aeruginosa*.

In agreement with previous research (Pedriali et al., 2010; Zaynab et al., 2010), the study results indicate that the high concentrations of crude PPE tested were not toxic to human cell lines until 30 h of exposure. The polyphenols are known to regulate different pathways; in detail, polyphenols protect HeLa cells against reactive oxygen species, and they increase pro-oxidant effects depending on the presence of iron or H_2_O_2_ in the medium (You et al., 2010).

Although there are well-documented antiproliferative effects of the phenolic compounds of pomegranate (Baradaran Rahimi et al., 2020; Gonzalez-Castillo et al., 2021), our experimental data report, for the first time, a protective effect on cellular systems with high concentrations of PPE. In line with this finding, a recent study has shown that pomegranate extract is able to mitigate and reduce the production of free radicals in endothelial cells after lipopolysaccharides exposure (Ciampi et al., 2020). However, further studies are needed to understand the antioxidant role of polyphenolic pomegranate extract on the cellular system.

Currently, it is known that the human aerobic metabolism leads to the generation of reactive oxygen species. The human body has multiple mechanistic, both enzymatic and non-enzymatic, antioxidant systems that can prevent oxidative damage. Endogenous antioxidant systems are not generally sufficient for protection against oxidative stress, and therefore, exogenous antioxidants are necessary to restrict free radical damage (Nabavi et al., 2012). The protective effects of some natural products against free radical-induced oxidative stresses have been surveyed in red blood cell membranes (Chaudhuri et al., 2007; Nabavi et al., 2012). In agreement with these data, PPE did not affect intracellular ROS production in human cells (Figure 4) and exerted an appreciable inhibitory activity of hemolysis with values ranging from 85 to 104% (Figure 5), suggesting a possible role as a natural antioxidant agent.

Although there is extensive knowledge on the *in vitro* antimicrobial activity of pomegranate extract, little is known about its antimicrobial activity in cellular infection models. To the best of our knowledge, we report, for the first time, a significant intracellular antibacterial property of PPE as highlighted by its bactericidal action against the staphylococcal reference strain and its bacteriostatic effect against MRSA strain 2 in the HeLa cell line.

Altogether, these findings demonstrate that PPE not only enhances the stability of eukaryotic plasma membranes but also can reach the intracellular environment revealing its antimicrobial action. Our results encourage the investigation and characterization of new biologically active molecules deriving from food industry by-products as a rich source of phenolic compounds with high antioxidant and antimicrobial properties. The obtained promising results based on the reuse of by-products of pomegranate against ESKAPE pathogens represent integrated solutions to promote human and animal health in a One Health approach.

In conclusion, although further *in vitro* and *in vivo* research studies are needed, our study suggests that PPE could be considered a therapeutic agent alone or in conjunction with standard antibiotics against challenging infections sustained by ESKAPE pathogens.

## Data availability statement

The raw data supporting the conclusions of this article will be made available by the authors, without undue reservation.

## Ethics statement

Ethical approval was not required for the studies on humans in accordance with the local legislation and institutional requirements because only commercially available established cell lines were used.

## Author contributions

ES: Investigation, Methodology, Writing – original draft. DS: Investigation, Methodology, Writing – original draft. GM: Formal analysis, Investigation, Writing – review & editing. MR: Investigation, Methodology, Writing – review & editing. LC: Formal analysis, Investigation, Writing – review & editing. MV: Funding acquisition, Supervision, Validation, Writing – review & editing. CaP: Resources, Supervision, Validation, Writing – review & editing. RC: Funding acquisition, Supervision, Validation, Visualization, Writing – original draft. ChP: Conceptualization, Funding acquisition, Supervision, Validation, Visualization, Writing – original draft. PS: Conceptualization, Funding acquisition, Supervision, Validation, Visualization, Writing – review & editing, Writing – original draft.
